# SKA3 Expression as a Prognostic Factor for Patients with Pancreatic Adenocarcinoma

**DOI:** 10.3390/ijms25105134

**Published:** 2024-05-09

**Authors:** Karolina Buchholz, Justyna Durślewicz, Anna Klimaszewska-Wiśniewska, Magdalena Wiśniewska, Maciej Słupski, Dariusz Grzanka

**Affiliations:** 1 Department of Clinical Pathomorphology, Faculty of Medicine, Collegium Medicum in Bydgoszcz, Nicolaus Copernicus University in Toruń, 85-094 Bydgoszcz, Poland; karolina.buchholz@cm.umk.pl (K.B.); justyna.durslewicz@cm.umk.pl (J.D.); d_grzanka@cm.umk.pl (D.G.); 2 Department of Histology and Embryology, Faculty of Medicine, Collegium Medicum in Bydgoszcz, Nicolaus Copernicus University in Toruń, 85-092 Bydgoszcz, Poland; 3 Department of Oncology and Brachytherapy, Faculty of Medicine, Collegium Medicum in Bydgoszcz, Nicolaus Copernicus University in Toruń, 85-796 Bydgoszcz, Poland; magdalena.wisniewska@cm.umk.pl; 4 Clinical Department of Oncology, Professor Franciszek Lukaszczyk Oncology Center in Bydgoszcz, 85-796 Bydgoszcz, Poland; 5 Department of General, Hepatobiliary and Transplant Surgery, Faculty of Medicine, Collegium Medicum in Bydgoszcz, Nicolaus Copernicus University in Toruń, 85-094 Bydgoszcz, Poland; maciej.slupski@cm.umk.pl

**Keywords:** pancreatic adenocarcinoma, SKA3, prognostic factor

## Abstract

The spindle and kinetochore-associated complex subunit 3 (SKA3) is a protein essential for proper chromosome segregation during mitosis and thus responsible for maintaining genome stability. Although its involvement in the pathogenesis of various cancer types has been reported, the potential clinicopathological significance of SKA3 in pancreatic ductal adenocarcinoma (PDAC) has not been fully elucidated. Therefore, this study aimed to assess clinicopathological associations and prognostic value of SKA3 in PDAC. For this purpose, in-house immunohistochemical analysis on tissue macroarrays (TMAs), as well as a bioinformatic examination using publicly available RNA-Seq dataset, were performed. It was demonstrated that SKA3 expression at both mRNA and protein levels was significantly elevated in PDAC compared to control tissues. Upregulated mRNA expression constituted an independent unfavorable prognostic factor for the overall survival of PDAC patients, whereas altered SKA3 protein levels were associated with significantly better clinical outcomes. The last observation was particularly clear in the early-stage tumors. These findings render SKA3 a promising prognostic biomarker for patients with pancreatic ductal adenocarcinoma. However, further studies are needed to confirm this conclusion.

## 1. Introduction

Pancreatic cancer is one of the most lethal malignant neoplasms worldwide. With almost as many deaths (466,003) as new cases (495,773) in 2020, it constitutes the seventh leading cause of cancer-related death globally [1]. Due to the asymptomatic initial course and rapid spread to surrounding organs, the disease is often diagnosed at the advanced stages. Difficulty in early detection, aggressive nature of pancreatic cancer, and relatively low effectiveness of treatment regimens result in a very dismal prognosis with a 5-year survival rate of approximately 6% (range 2–9%) [2].

Pancreatic ductal adenocarcinoma (PDAC) is the most common type of pancreatic cancer accounting for more than 90% of cases [3]. Its development and progression is a multi-step process in which unstable genome and defective DNA repair pathways play a key role. It was observed that one of the earliest molecular changes in PDAC precursors is telomere shortening which, as a source of chromosomal instability, may lead to missegregation during mitosis and subsequent progressive accumulation of chromosomal abnormalities. Furthermore, various genetic alterations involved in the progression from early-stage neoplasia to PDAC have been described, including *KRAS*, *CDKN2A*, *TP53*, and *SMAD4* mutations [4]. However, despite strenuous efforts made by scientists around the world, useful diagnostic and prognostic markers of PDAC as well as an effective treatment strategy have not been established so far. Therefore, considering early molecular events observed during PDAC development, we chose spindle and kinetochore-associated complex subunit 3 (SKA3) involved in chromosome segregation to determine its clinical value in examined cancer.

SKA3 is a member of the SKA complex located in the kinetochore outer layer [5]. This protein in cooperation with the NDC80 complex stabilizes the kinetochore-microtubule interaction and silences the spindle assembly checkpoint after proper metaphase chromosome alignment. In this manner, it regulates mitotic exit during cell division [6,7,8]. Previous studies showed that SKA3 is aberrantly expressed in numerous tumors and thereby associated with cancer progression and poor prognosis of patients [9,10,11,12,13]. However, the relationship between SKA3 and PDAC has not been elucidated.

Given the above, the present study was designed to analyze clinicopathological associations and prognostic value of SKA3 in PDAC. For this purpose, the expression of SKA3 protein was evaluated using immunohistochemically stained tissue macroarrays (TMAs), while *SKA3* gene expression data were downloaded from publicly available databases. Furthermore, functional enrichment analysis and protein-protein interaction network were used to predict the biological significance of *SKA3* in pancreatic adenocarcinoma (PAAD).

## 2. Results

### 2.1. SKA3 Protein Expression in PDAC and Non-Cancerous Adjacent Tissue: Association with Patients’ Characteristics

**Figure 1:**
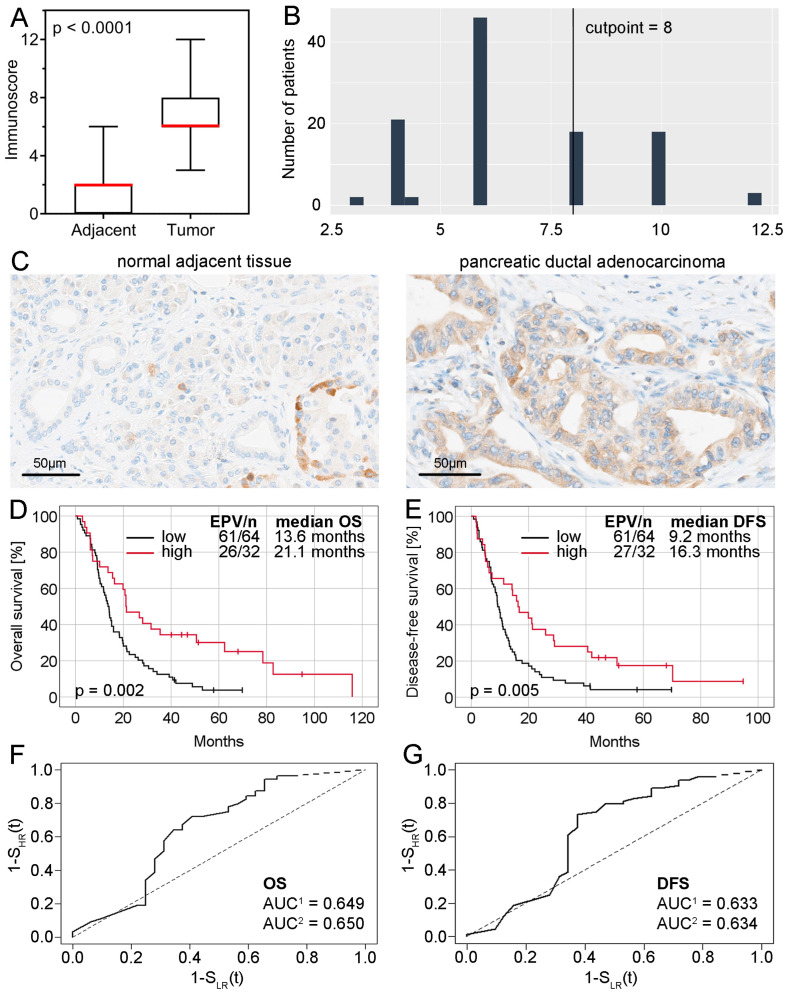
SKA3 protein expression and its correlation with PDAC patient survival. (**A**) Comparison of SKA3 protein levels in tumor and non-cancerous adjacent tissues of PDAC patients. The error bars present the range from minimum to maximum values of data. The medians for both sets of values have been marked in red. (**B**) SKA3 IRS distribution with a marked cut-off point established using the cutp function of the Evaluate Cutpoints software (Cp = 8). (**C**) Representative images of immunohistochemical expression of SKA3 in pancreatic ductal adenocarcinoma and normal adjacent tissue (control). Original magnification 20×. (**D**) Kaplan-Meier curves assessing the relationship between SKA3 protein expression and OS of PDAC patients in the TMA cohort (n = 96); (**E**) Kaplan-Meier curves assessing the relationship between SKA3 protein expression and DFS of PDAC patients in the TMA cohort (n = 96); (**F**,**G**) corresponding prognostic ROC curves extrapolated with noninformative (AUC^1^) and optimistic (AUC^2^) assumptions. Abbreviations: AUC—area under the curve, DFS—disease-free survival, EPV—events per variable, OS—overall survival, ROC—receiver operating characteristic.

Immunoreactivity assessed in TMAs was restricted to the cytoplasm both in cancer and non-tumor cells. Simultaneously, levels of SKA3 were significantly higher in PDAC in comparison to normal appearing adjacent pancreatic ductal epithelium (*p* < 0.0001; Figure 1A). As a result of the immunoreactive score (IRS) dichotomization (Figure 1B), a high level of SKA3 was found in 39 (35.5%) cases of PDACs, whereas a low level was observed in 71 (64.5%) PDAC tumors and all 71 (100%) applied control samples. Representative images of immunohistochemical staining are presented in Figure 1C. Furthermore, the expression status of SKA3 was not associated with any of the analyzed clinicopathological features (*p* > 0.05; Table S1).

### 2.2. Association between the SKA3 Protein Expression and PDAC Patients’ Survival (n = 96)

Fourteen patients in the TMA cohort were confirmed to have died due to postoperative complications. These cases were excluded, and further survival analyses were performed on a study group consisting of 96 patients. Median overall survival (OS) time and disease-free survival (DFS) time in this group of patients were 15.1 (95% CI 12.9–17.3) and 10.6 (95% CI 8.1–13.1) months, respectively. To explore the association between SKA3 protein expression and PDAC patient survival, Kaplan-Meier curves and log-rank test were used. It was observed that patients whose PDAC tumors presented high levels of SKA3 had significantly better OS and DFS than those expressing low levels of the analyzed protein (OS: 21.1 months vs. 13.6 months, log-rank *p* = 0.002, Figure 1D; DFS: 16.3 months vs. 9.2 months, log-rank *p* = 0.005, Figure 1E).

**Figure 2:**
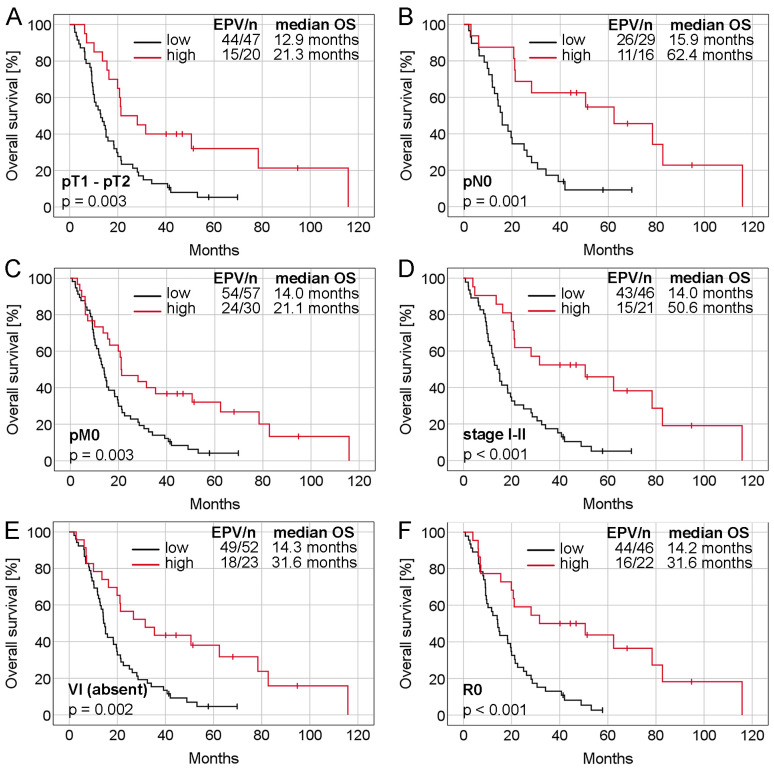
Kaplan-Meier curves presenting the overall survival depending on SKA3 protein expression in different subgroups of PDAC patients. Analyzed subgroups: (**A**) pT1-pT2, (**B**) pN0, (**C**) pM0, (**D**) TNM stage I–II, (**E**) absence of VI and (**F**) resection margin R0. Abbreviations: EPV—events per variable, OS—overall survival, VI—vascular invasion.

To further determine the prognostic importance of SKA3 protein expression, prognostic receiver operating characteristic (ROC) curves and Cox regression analyses were carried out. The first of these was performed to evaluate the area under the curve (AUC) and thus the probability of shorter OS and DFS time for PDAC patients in the high-risk (low expression) group compared to those in the low-risk (high expression) group. The AUC for OS was 0.649 (noninformative scenario) and 0.650 (an optimistic scenario), therefore risk was estimated as 65% (Figure 1F). In the case of DFS, the AUC was 0.633 (noninformative scenario) and 0.634 (an optimistic scenario) with a risk of 63% (Figure 1G). In unadjusted Cox proportional hazards regression, SKA3 expression level was significantly associated with OS and DFS (OS: HR = 0.47, 95% CI 0.29–0.77, *p* = 0.003; DFS: HR = 0.52, 95% CI 0.32–0.83, *p* = 0.006; Table 1). Multivariable regression analysis confirmed this factor as an independent predictor of both OS and DFS in PDAC patients (OS: adjusted HR = 0.40, 95% CI 0.24–0.67, *p* < 0.001; DFS: adjusted HR = 0.48, 95% CI 0.29–0.79, *p* = 0.004; Table 1). Further analysis revealed that the expression of SKA3 protein in stages I–II (adjusted HR = 0.28, 95% CI 0.14–0.59, *p* < 0.001) but not in stage III or stages III–IV (*p* > 0.05 in univariable Cox regression analyses, data not shown) constituted an independent favorable prognostic factor for OS (Table 2). A significant relationship between SKA3 overexpression and longer median OS time of PDAC patients was also noted in the following subgroups: T1–T2 tumors, N0, M0, stages I–II, VI-negative and resection margin-negative (log-rank test *p* < 0.05, Figure 2). Collectively, these findings show that SKA3 expression has prognostic significance in PDAC, especially in early-stage disease, for which the contribution of SKA3 is even greater.

### 2.3. SKA3 mRNA Expression in PDAC and Normal Pancreatic Tissue: Association with Patients Characteristics and Genome Instability Parameters

**Figure 3:**
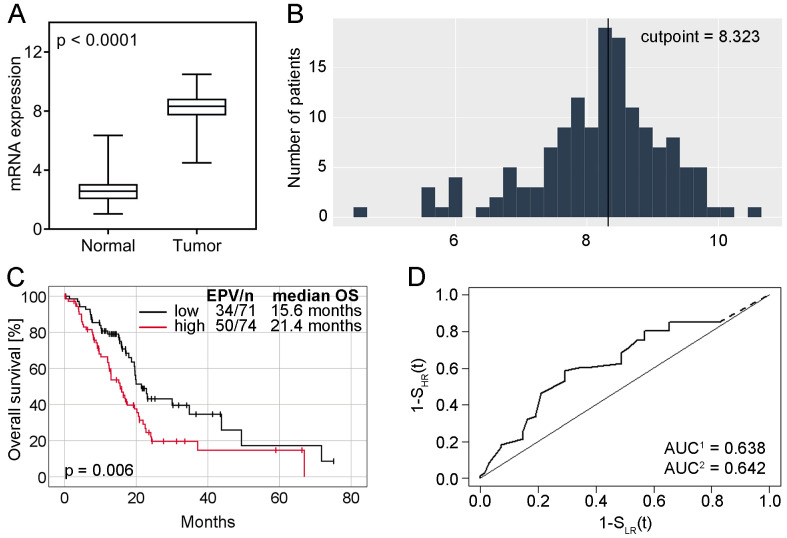
*SKA3* mRNA expression and its correlation with PDAC patients survival. (**A**) Comparison of *SKA3* mRNA expression levels in tumor and normal tissue samples of PDAC patients. The error bars present the range from minimum to maximum values of data. (**B**) *SKA3* expression levels distribution with a marked optimal cut point established with the Evaluate Cutpoints software (Cp = 8.323). (**C**) Assessment of the relationship between *SKA3* expression and OS of PDAC patients. Kaplan-Meier curve comparing cases of TCGA cohort with high and low *SKA3* expression (n = 145); (**D**) corresponding prognostic ROC curve extrapolated with noninformative (AUC^1^) and optimistic (AUC^2^) assumptions. Abbreviations: AUC—area under the curve, EPV—events per variable, OS—overall survival, ROC—receiver operating characteristic, TCGA—The Cancer Genome Atlas.

Analysis of RNA-sequencing-based data showed that the expression levels of *SKA3* were significantly higher in PDACs compared to normal pancreatic tissues (*p* < 0.0001; Figure 3A). According to the optimal cutpoint established with the Evaluate cutpoint software (Figure 3B), upregulation of *SKA3* was observed in 74 (51%) PDACs, while downregulation in the other 71 (49%). In the case of normal pancreatic tissue, all 153 (100%) samples demonstrated low expression of the analyzed gene. No significant associations were found between *SKA3* expression and clinicopathological traits (*p* > 0.05; Table S2). In the context of genome instability parameters, *SKA3* expression correlated with the fraction of genome altered (r = 0.61, *p* < 0.0001), aneuploidy score (r = 0.49, *p* < 0.0001), mutation count (r = 0.39, *p* < 0.0001) and MSIsensor score (r = 0.19, *p* = 0.02), but not with and MSI MANTIS score (r = 0.11, *p* = 0.19).

### 2.4. Association between the SKA3 mRNA Expression and PDAC Patients’ Survival

In Kaplan-Meier survival analysis, high expression of *SKA3* correlated with significantly shorter median OS time of PDAC patients in comparison to those with *SKA3* low expression (15.6 months vs. 21.4 months, log-rank test *p* = 0.006, Figure 3C). The area under the prognostic ROC curve was 0.638 (a noninformative scenario) and 0.642 (an optimistic scenario): the probability of earlier dying of *SKA3* overexpressors was 64% (Figure 3D). Univariable Cox regression analysis demonstrated that *SKA3* upregulation (HR = 1.84, 95% CI 1.18–2.86, *p* = 0.007, Table 3) was related to an unfavorable survival rate. Multivariable Cox proportional hazard model revealed *SKA3* mRNA expression (adjusted HR = 2.13, 95% CI 1.36–3.34, *p* < 0.001; Table 3) as independent poor prognostic factor for OS.

### 2.5. Functional Enrichment Analysis

**Figure 4:**
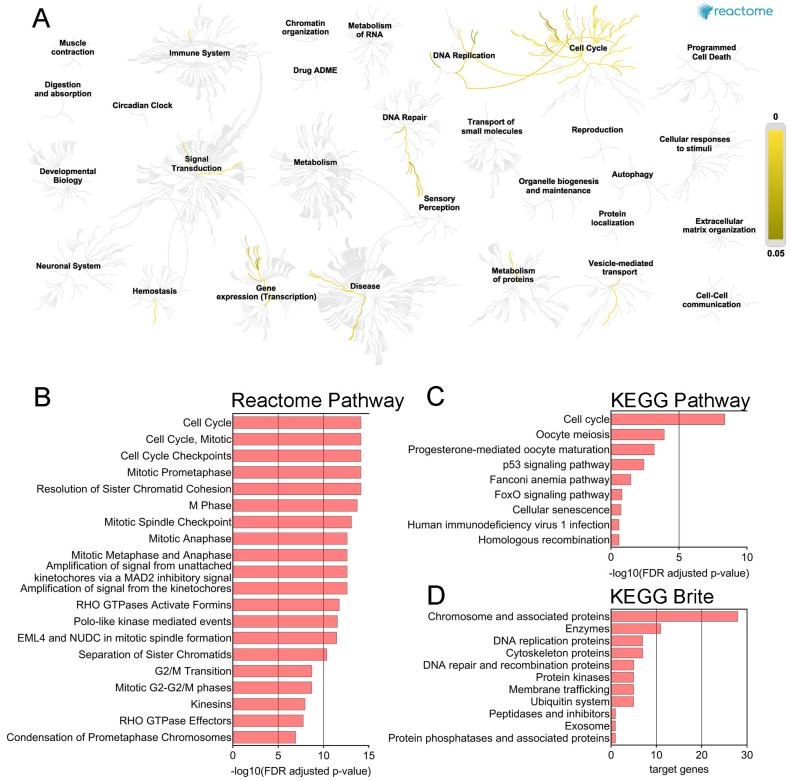
Functional enrichment analysis for *SKA3* and its 50 interaction partners in PAAD. (**A**) The Reactome Pathway hierarchy panel. (**B**) Bar chart of the top 20 Reactome pathways based on the enrichment scores [−log10 (FDR adjusted *p*-value)]. (**C**) Bar chart for all KEGG Pathways based on the enrichment scores [−log10 (FDR adjusted *p*-value)]. (**D**) Bar chart for all terms in BRITE functional hierarchies. The horizontal axis represents the number of genes enriched in KEGG Brite terms represented on the vertical axis.

The Reactome Pathway hierarchy panel for *SKA3* and its 50 interaction partners in PAAD is presented in Figure 4A. The analysis demonstrated that imputed genes were primarily associated with cell cycle, cell cycle mitotic, cell cycle checkpoints, mitotic prometaphase, and resolution of sister chromatid cohesion (Figure 4B, Table S3). KEGG pathway analysis revealed that the gene set was significantly enriched in five pathways: cell cycle, oocyte meiosis, progesterone-mediated oocyte maturation, p53 signaling pathway, and Fanconi anemia pathway (Figure 4C, Table S4). According to KEGG Brite studies, there was a preponderance of genes representing chromosome and associated proteins, enzymes, DNA replication proteins, and cytoskeleton proteins (Figure 4D).

**Figure 5:**
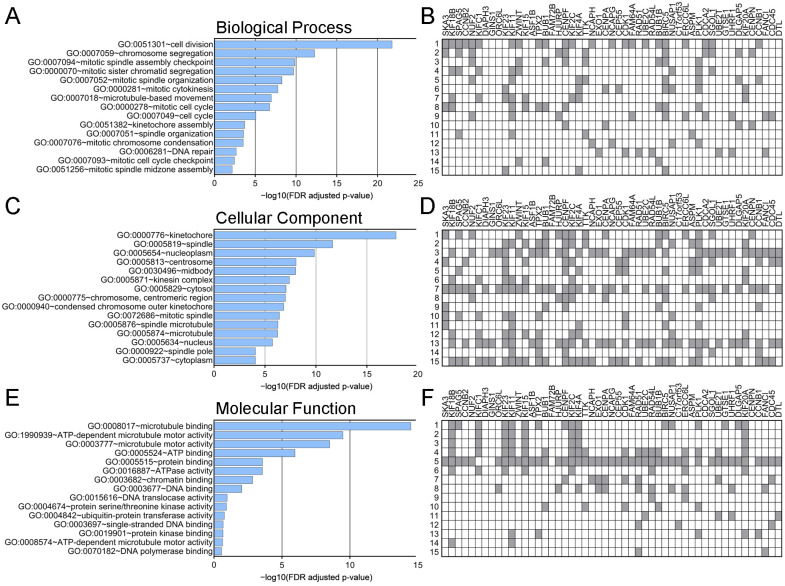
Gene Ontology enrichment analysis for *SKA3* and its 50 interaction partners in PAAD. Bar charts showing the top enriched terms for (**A**) biological process, (**C**) cellular component, and (**E**) molecular function ranked by [−log10 (FDR adjusted *p*-value)]. (**B**,**D**,**F**) Heatmaps based on the target genes.

Finally, GO functional enrichment analysis indicated that *SKA3* and co-upregulated genes were significantly involved in 19 GO terms for biological process (BP), 25 GO terms for cellular components (CC), and 8 GO terms for molecular functions (MF; Table S5). As shown in Figure 5, the most enriched ontology terms were cell division (BP, GO:0051301; Figure 5A,B), kinetochore (CC, GO:0000776; Figure 5C,D), and microtubule binding (MF, GO:0008017; Figure 5E,F).

### 2.6. Protein-Protein Interaction (PPI)

**Figure 6:**
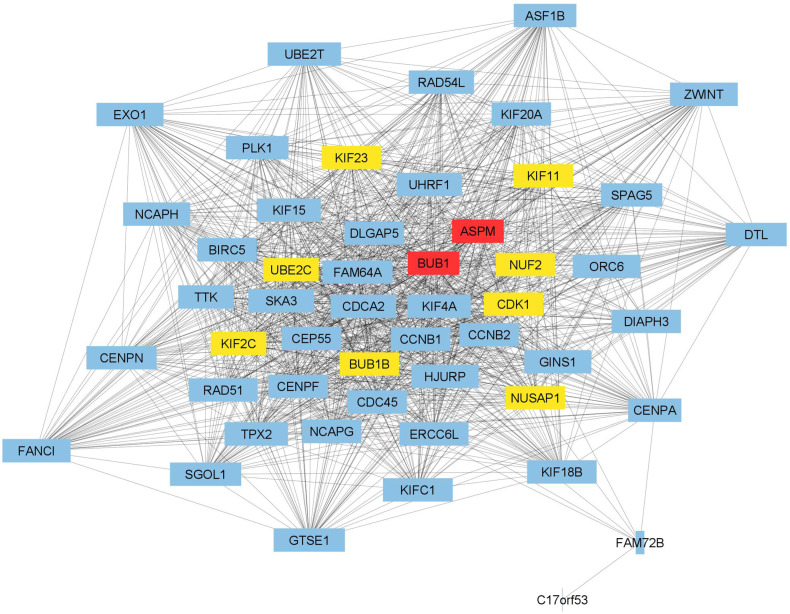
The PPI network of *SKA3* and its top 50 neighboring genes in PAAD. The nodes represent analyzed genes, while the interactions between them are shown as edges. The clustering coefficient of PPI network is visualized by the width of nodes. The top 10 hub genes established based on the degree score are distinguished in a yellow to red gradient with the highest values for red color. Other genes are marked in blue.

The STRING database and Cytoscape software (version 3.9.1) were used to construct and visualize a PPI network of *SKA3* and its 50 neighboring genes identified as most correlated in PAAD (Figure 6). It was noted 51 nodes and 1111 edges in the PPI network. PPI enrichment *p* value was lower than 1.0 × 10^−16^, and the average local clustering coefficient was 0.948. Detailed information on PPI network parameters computed with NetworkAnalyzer Cytoscape plugin was depicted in Table S6. The top 10 hub genes determined using the CytoHubba Cytoscape plugin and based on the degree score were presented as colored nodes (Figure 6).

## 3. Discussion

In the present study, the evaluation of the SKA3 expression status concerning patients’ characteristics and clinical outcomes was performed to investigate the potential prognostic value of this factor in PDAC. For this purpose, institutional TMAs and publicly available RNA-seq datasets were used. Finally, the PPI network for *SKA3* and its 50 neighbors was constructed and functionally annotated.

Our research demonstrated that *SKA3* expression was significantly upregulated in PDAC tumors compared to control samples. Several mechanisms might explain the observed differences. According to Li et al., one factor affecting *SKA3* expression in PAAD is DNA methylation. Another possible cause could be gene alterations, including single nucleotide variants. It has been shown that the frequency of these deleterious mutations in PAAD can be as high as 1% [14]. Liu et al. also reported significant differences in *SKA3* mRNA expression between PDAC tumors and control tissues, similar to our findings. To the best of our knowledge, this is the only study to date reporting the prognostic value of the analyzed gene in ductal type of pancreatic cancer [15]. Authors additionally demonstrated that *SKA3* overexpression was more frequently detected in poorly differentiated and undifferentiated tumors (G3–G4) compared to well and moderately differentiated (G1–G2) ones. However, this observation was noted only for GSE62452 dataset. In the The Cancer Genome Atlas (TCGA) cohort, no significant relationships between mRNA expression and the examined clinical features were found [15], which is consistent with our results. Upregulation of *SKA3* and associated cancer progression have also been reported in other tumors, including lung adenocarcinoma [16], prostate cancer [17], breast cancer [18], glioma [19], kidney renal papillary cell carcinoma [13], skin cutaneous melanoma [20] and bladder cancer [21].

The Kaplan-Meier estimation performed by Liu et al. to compare OS time in the high and low *SKA3* expression groups showed significant and borderline significant differences for GEO and TCGA datasets, respectively [15]. Shorter OS time was noticeably associated with the upregulation of *SKA3*, which is aligned with our results obtained for the TCGA cohort. As the main goal of the present report was to assess the prognostic value of *SKA3* in PDAC, we also performed Cox regression analyses. These demonstrated that increased expression of *SKA3* was correlated with significantly shorter patient survival, and after adjusting for confounding variables, it remained an independent unfavorable prognostic factor for OS of PDAC patients. Our findings align with observations from other studies indicating that *SKA3* overexpression predicts poor prognosis and is significantly associated with OS in PDAC [15] as well as with OS, disease-specific survival, and disease-free interval in PAAD [14].

To gain deeper insights into the mechanisms underlying the prognostic value of *SKA3* in PAAD, we first identified genes that correlate with *SKA3* expression and then constructed a PPI network to find genes with a high degree of interaction and thus potentially related to the development of PAAD. In our PPI network, the nodes with the highest degree centrality were ASPM and BUB1. It has been demonstrated that ASPM plays a role in regulating Wnt signaling and cancer stemness in PDAC. ASPM isoform I (ASPM-iI), which is localized in the cytoplasm, interacts with disheveled-2 and active β-catenin, key components of the Wnt pathway. This interaction drives Wnt signaling, contributing to cancer stemness and tumorigenicity. In turn, ASPM isoform II (ASPM-iII), primarily located in the nucleus, regulates the cell cycle by interacting with cyclin E [22]. The second hub gene, *BUB1*, encodes a serine/threonine protein kinase that is a crucial component of the spindle assembly checkpoint. The interaction between *BUB1* and *SKA3* observed in our results has also been confirmed in in vitro studies. Namely, it has been demonstrated that reducing *SKA3* expression in HeLa cell line causes mitotic arrest, accompanied by a strong accumulation of the checkpoint protein BUB1 at kinetochores, particularly in cells with misaligned chromosomes [23]. In studies conducted on pancreatic cancer cells, *BUB1* was identified as a promoter of cell proliferation, migration and gemcitabine resistance [24]. Both *ASPM* and *BUB1* interact with *UBE2C*, *KIF23*, *KIF2C*, *KIF11*, *NUF2*, *CDK1*, *BUB1B* and *NUSAP1*, each of which has previously been associated with the initiation and/or progression of PAAD [25,26,27,28,29,30,31,32]. The results of our functional enrichment analysis suggest that *SKA3* is correlated with genes that promote proliferation, as well as those responsible for ensuring the high fidelity of chromosome segregation. High expression of *SKA3* in PAAD may therefore lead to uncontrolled cell proliferation and the development of aneuploidy due to disrupted chromosome segregation, ultimately worsening patient prognosis.

To the best of our knowledge, the present study is the first to demonstrate the prognostic value of SKA3 protein in PDAC. We showed that a high expression of this factor was significantly more often in PDAC tumors than in non-tumor adjacent tissues, but simultaneously, no correlations between SKA3 expression and analyzed clinicopathological traits were noted. Attempts to elucidate the role of the SKA3 in cancers other than PDAC have yielded several conclusions. Hou et al. demonstrated that the knockdown of SKA3 in hepatocellular carcinoma inhibits proliferation and tumor invasion both in vitro and in vivo by regulating CDK2/P53 phosphorylation [33]. In lung adenocarcinoma, SKA3 was identified as an oncogene promoting cell growth and migration by PLK1-mediated SKA3 phosphorylation [34] and stimulating metastasis through the activation of PI3K-AKT signaling [16]. Similarly in cervical cancer (CC), the PI3K-AKT pathway was revealed to be involved in promoting the proliferation and migration of CC cells overexpressing SKA3 [35]. Additionally, Zhang et al. found that SKA3 regulates DUSP2 and thus activates the MAPK/ERK pathway in gastric cancer (GC). As a result, GC cells are stimulated to proliferation and EMT and this in turn leads to invasion and even peritoneal metastasis [36].

The next step of the present research was to assess the prognostic value of SKA3 protein in PDAC. It is considered that pancreatic cancer resection remains one of the most challenging surgical procedures, still burdened with a high percentage of postoperative complications affecting the survival time of patients [37]. In this discipline, the key roles are played by appropriately qualifying the patient for surgical treatment, the surgical technique, and the ability to administrate postoperative complications [38]. Wegner et al. identified also preoperative treatment, increasing age, higher comorbidity score, lower case volume, lower income, and type of surgery as significant negative predictors of 30-day postoperative mortality [39]. Therefore, to eliminate the possible impact of postoperative deaths on the prognostic value assessment of SKA3 protein, patients for whom postoperative complications were confirmed as the cause of death were excluded from survival analyses. The Kaplan-Meier estimation and log-rank test demonstrated that elevated levels of SKA3 were associated with noticeably longer OS and DFS. A significant relationship between high SKA3 expression and better OS time of PDAC patients was also noted in the following subgroups: T1–T2 tumors, M0, N0, TNM stage I–II, absence of VI, and resection margin R0. Cox regression analysis additionally confirmed SKA3 protein to be an independent predictor of OS and DFS. For OS endpoint, the last observation was found both in the full TMA cohort and early-stage tumors (TNM stage I–II) analyzed separately.

The obtained results indicate high *SKA3* mRNA expression and low SKA3 protein level as unfavorable prognostic factors in PDAC. The lack of correlation between these two levels of biological information is not surprising. Many examples of similar discrepancies which can be explained by posttranscriptional and posttranslational modifications have been presented in the literature so far [40,41]. However, the regulation of gene expression is not the only possible cause of the discussed discordances. They can also result from tumor heterogeneity or even the specificity of the dataset (e.g., distribution of clinical data, sample size). Inference based only on mRNA expression data or protein expression level is questionable and therefore parallel studying of both parameters is a best practice, crucial for a better understanding of gene and protein networks and thus the functional significance of a pathway. In this context, a significant limitation of the present study is that the tumors examined for protein expression are not the same as those examined in mRNA analysis. Finally, especially due to the unique pathobiology of PDAC, performing in vitro experiments explaining the role of SKA3 in this cancer would be a valuable supplement to the obtained results.

## 4. Conclusions

To sum up, the present study is the second to assess the prognostic value of *SKA3* gene in PDAC and the first to investigate SKA3 protein in this deadly disease. Firstly, we demonstrated that the upregulation of mRNA constituted an independent unfavorable prognostic factor for the overall survival of PDAC patients. Thereafter, we found that high protein levels were associated with significantly better clinical outcomes, especially in the early stages of cancer. The above discrepancy highlights the need for further investigation, including larger cohorts and in vitro experiments, to clarify the role of SKA3 in PDAC pathogenesis.

## 5. Materials and Methods

### 5.1. Tissue Material and Clinicopathological Data

Tissue samples, including tumors (n = 110) and adjacent non-cancerous tissues (n = 71), were obtained from PDAC patients undergoing pancreatic resection between 2009 and 2020 at the Department of General, Hepatobiliary and Transplant Surgery of the A. Jurasz University Hospital No. 1 in Bydgoszcz (Poland). The TNM classification of all PDAC specimens was determined based on the eighth edition of the American Joint Committee on Cancer staging system. The study group consisted of the cohort of patients described in previous articles [42,43] supplemented with additional cases. Inclusion criteria were as follows: (I) pathological diagnosis of PDAC; (II) availability of complete clinical data on age, sex, grading, pT status, pN status, pM status, TNM stage, vascular invasion, perineural invasion, resection margin, and chemotherapy. Two different endpoints were used in the study: OS (defined as the time from surgery to death from any cause) and DFS (defined as the time from surgery to any recurrence or death). Follow-up data collection concluded in September 2023. The research protocol was approved by the Bioethics Committee at Collegium Medicum in Bydgoszcz of Nicolaus Copernicus University in Toruń (no. 342/2020) and performed according to the guidelines of the Declaration of Helsinki.

### 5.2. Immunochistochemistry

Immunohistochemical staining was performed on TMAs according to the previously described method [42]. Whole tissue sections at thickness of 4-μm obtained by sectioning recipient paraffin blocks were labeled with primary rabbit polyclonal anti-SKA3 antibody (1:1500, 32 min; cat. no: PA558722, Thermo Fisher Scientific, Waltham, MA, USA) using BenchMark ULTRA system (Roche Diagnostics/Ventana Medical Systems, Tucson, AZ, USA). Antigen-antibody complexes were visualized with ultraView Universal DAB Detection Kit (Roche Diagnostics/Ventana Medical System, Tucson, AZ, USA). Positive control was performed based on data available in The Human Protein Atlas and antibody datasheet provided by the manufacturer, while the negative control was obtained by omitting the primary antibody.

The immunohistochemical expression of SKA3 protein was evaluated by two investigators, including the senior pathologist (D.G.), under a multi-headed microscope (Olympus, Tokyo, Japan) at 20× original objective magnification. Protein levels were assessed within PDAC and normal duct epithelium using the IRS which considers both the intensity of staining (IS; 0: negative, 1: weak, 2: moderate, 3: strong) and the percentage of positively stained cells (PS; 0: <5%; 1: 6–25%; 2: 26–50%; 3: 51–75%; 4: >75%). The final immunoscore ranging from 0 to 12 was calculated by multiplying IS and PS. Results obtained for all analyzed cases were dichotomized into low and high expression groups based on the optimal cutpoint determined using the cutp function of the Evaluate Cutpoints software (<8.0; ≥8.0, respectively) [44].

### 5.3. RNA-Sequencing Data

RNA-sequencing (RNA-seq) data normalized via DESeq2 method for 178 PAAD tumors and 153 normal tissue samples were retrieved from TCGA and Genotype-Tissue Expression databases, respectively. The University of California Santa Cruz Xena Browser was tilized for this purpose [45]. Clinicopathological data of patients with PAAD were obtained from the cBio Cancer Genomics Portal [46]. Only patients with a ductal type of tumor and those for whom clinical data were available in cBioPortal were included in the study. Additionally, one patient with a survival time of 0 months was excluded from the analyses, resulting in a final study group of 145 patients. To minimize the bias due to missing data (Table S7), all survival analyses for the TCGA cohort were performed on dataset subjected to multiple imputation (MI; n = 10). Due to a substantial amount of missing data on distant metastasis (pM), this variable was not included in the MI procedure and further survival analyses. *SKA3* mRNA expression data were divided into low (<8.323) and high (≥8.323) expression groups based on the optimal cutpoint established with the cutp function of the Evaluate Cutpoints software [44]. The follow-up and median OS time of PDAC patients were 23.5 (95% CI 20.5–26.5) and 19.5 (95% CI 17.0–21.9) months, respectively.

### 5.4. Functional Enrichment Analysis

The University of Alabama at Birmingham Cancer data analysis Portal (UALCAN) [47] was searched to identify the top 50 genes positively correlated with *SKA3* in PAAD tissue (Figure S1). These were used to perform FEA and to construct the PPI network afterward. Pathway analysis and visualization were conducted with the Reactome pathway database [48], while the functional hierarchies of imputed genes were explored using The Kyoto Encyclopedia of Genes and Genomes (KEGG) Biomolecular Relations in Information Transmission and Expression (BRITE) database [49]. Gene Ontology (GO) and KEGG pathway enrichment analyses were carried out using the Database for Annotation, Visualization, and Integrated Discovery (DAVID) [50]. Enriched GO terms were classified into three categories: biological process (BP), cellular component (CC), and molecular function (MF). For all analyses, False Discovery Rate (FDR) adjusted *p*-value < 0.05 (q-value) was considered statistically significant.

### 5.5. Construction of the Protein-Protein Interaction Network

A PPI network involving *SKA3* and its 50 most relevant neighboring genes was constructed based on data retrieved from Search Tool for the Retrieval of Interacting Genes/Proteins (STRING) database [51]. To visualize the PPI network, Cytoscape software (version 3.9.1) [52] along with NetworkAnalyzer and CytoHubba plugins were used.

### 5.6. Statistical Analysis

Statistical analyses were carried out with the GraphPad Prism (version 8.0, GraphPad Software, San Diego, CA, USA), SPSS software packages (version 28.0, IBM Corporation, Armonk, NY, USA) or survival and survminer R packages (version 1.3.1093 of RStudio, Vienna, Austria). The Shapiro-Wilk test was used to verify data normality. Differences between continuous variables were analyzed by the Mann-Whitney test, whereas the strength and direction of association between two ranked variables were measured using Spearman’s correlation coefficient (r). The chi-square or Fisher’s exact test was performed to assess the interrelation of categorized SKA3 expression data and patient clinicopathological characteristics. Differences in survival time between the high- and low-risk groups were estimated using the Kaplan–Meier curves and tested for significance by the log-rank test. Prognostic ROC curves (according to the method described by Combescure et al. [53]) and Cox proportional hazards regression models were used to predict the prognostic value of SKA3 levels in the analyzed cancer. To test for the proportional-hazards (PH) assumption, graphical diagnostics based on the scaled Schoenfeld residuals and plot log(-log(S(t))) vs. t were performed. In cases where the PH assumption did not hold, Cox regression models were built with time-dependent covariates. A value of *p*  <  0.05 was considered statistically significant.

## Figures and Tables

**Table 1:** Univariable and multivariable analyses of prognostic indicators by Cox regression model in the TMA cohort (n = 96).

Variable	EPV/n	Univariable Analysis				Multivariable Analysis ^#^			
		HR	95% CI		*p*	HR	95% CI		*p*
			Lower	Upper			Lower	Upper	
**Overall survival**									
SKA3 (low vs. high)	61/64_26/32	0.47	0.29	0.77	**0.003**	0.40	0.24	0.67	**<0.001**
Age (≤70 vs. >70)	70/79_17/17	2.45	1.39	4.30	**0.002**	0.89	0.43	1.85	0.75
Sex (female vs. male)	48/53_39/43	0.89	0.58	1.37	0.61	-	-	-	-
Grade (G1 vs. G2–G3)	6/7_81/89	1.27	0.55	2.92	0.57	-	-	-	-
pT (T1 vs. T2–T4)	13/18_74/78	1.77	0.98	3.21	0.06	-	-	-	-
pN (absent vs. present)	37/45_50/51	2.32	1.47	3.65	**<0.001**	-	-	-	-
pM (absent vs. present)	78/87_9/9	1.81	0.90	3.66	0.10	-	-	-	-
TNM stage (I–II vs. III–IV)	58/67_29/29	2.46	1.53	3.96	**<0.001**	3.15	1.85	5.37	**<0.001**
VI (absent vs. present)	67/75_20/21	2.07	1.24	3.45	**0.006**	2.20	1.24	3.89	**0.007**
PNI (absent vs. present)	15/19_72/77	1.71	0.97	3.03	0.06	-	-	-	-
R (R0 vs. R1)	60/68_27/28	1.60	1.00	2.55	**0.049**	1.11	0.68	1.82	0.68
CTX (no vs. yes)	13/13_74/83	0.25	0.14	0.47	**<0.001**	0.16	0.07	0.37	**<0.001**
**Disease-free survival**									
**Variable**	**EPV/n**	**Univariable analysis**				**Multivariable analysis ^#^**			
SKA3 (low vs. high)	61/64_27/32	0.52	0.32	0.83	**0.006**	0.48	0.29	0.79	**0.004**
Age (≤70 vs. >70)	71/79_17/17	1.90	1.10	3.28	**0.02**	0.80	0.37	1.72	0.56
Sex (female vs. male)	49/53_39/43	0.96	0.63	1.46	0.84	-	-	-	-
Grade (G1 vs. G2–G3)	7/7_81/89	0.99	0.45	2.15	0.98	-	-	-	-
pT (T1 vs. T2–T4)	13/18_75/78	2.01	1.11	3.64	**0.02**	-	-	-	-
pN (absent vs. present)	37/45_51/51	1.92	1.43	2.58	**<0.001**	-	-	-	-
pM (absent vs. present)	79/87_9/9								
pM		0.28 ^T^	0.04	2.03	0.21	-	-	-	-
pM*T_COV_		1.29 ^T^	1.04	1.61	**0.02**	-	-	-	-
TNM stage (I–II vs. III–IV)	59/67_29/29	3.02	1.83	4.97	**<0.001**	3.58	2.07	6.19	**<0.001**
VI (absent vs. present)	68/75_20/21	1.95	1.17	3.26	**0.01**	1.90	1.08	3.35	**0.03**
PNI (absent vs. present)	15/19_73/77	1.62	0.93	2.85	0.09	-	-	-	-
R (R0 vs. R1)	61/68_27/28	1.41	0.89	2.24	0.15	-	-	-	-
CTX (no vs. yes)	13/13_75/83	0.30	0.17	0.56	**<0.001**	0.18	0.08	0.44	**<0.001**

Abbreviations: CI—confidence interval, CTX—chemotherapy, EPV—events per variable, HR—hazard ratio, VI—vascular invasion, PNI—perineural invasion, R—resection margin, TMA—tissue macroarray. Significant *p*-values (*p* < 0.05) are indicated in bold. ^#^ Final result of a multivariable Cox regression model built of variables with *p*-value < 0.05 in univariable analysis. ^T^ HR for time-dependent variable.

**Table 2:** The univariable and multivariable Cox regression models in TNM stage I–II PDAC patients (TMA cohort, n = 67).

Variable	EPV/n	Univariable Analysis TNM Stage I–II				Multivariable Analysis ^#^ TNM Stage I–II			
		HR	95% CI		*p*	HR	95% CI		*p*
			Lower	Upper			Lower	Upper	
SKA3 (low vs. high)	43/46_15/21	0.31	0.16	0.61	**<0.001**	0.28	0.14	0.59	**<0.001**
Age (≤70 vs. >70)	46/55_12/12	3.16	1.58	6.35	**0.001**	1.19	0.45	3.10	0.73
Sex (female vs. male)	32/37_26/30	0.84	0.50	1.43	0.53	-	-	-	-
Grade (G1 vs. G2–G3)	4/5_54/62	1.27	0.46	3.53	0.65	-	-	-	-
pT (T1 vs. T2–T3)	10/15_48/52	1.71	0.86	3.39	0.13	-	-	-	-
pN (N0 vs. N1)	32/40_26/27	2.17	1.26	3.75	**0.005**	2.37	1.30	4.31	**0.005**
TNM stage (I vs. II)	25/32_33/35	1.63	0.96	2.76	0.07	-	-	-	-
VI (absent vs. present)	46/54_12/13	2.14	1.12	4.12	**0.02**	2.95	1.40	6.20	**0.004**
PNI (absent vs. present)	8/12_50/55	2.34	1.08	5.08	**0.03**	1.67	0.74	3.79	0.22
R (R0 vs. R1)	42/50_16/17	1.76	0.97	3.19	0.06	-	-	-	-
CTX (no vs. yes)	12/12_46/55	0.18	0.09	0.37	**<0.001**	0.15	0.06	0.42	**<0.001**

Abbreviations: CI—confidence interval, CTX—chemotherapy, EPV—events per variable, HR—hazard ratio, VI—vascular invasion, PNI—perineural invasion, R—resection margin, TMA—tissue macroarray. Significant *p*-values (*p* < 0.05) are indicated in bold. ^#^ Final result of a multivariable Cox regression model built of variables with *p*-value < 0.05 in univariable analysis.

**Table 3:** Univariable and multivariable analyses of prognostic indicators by Cox regression model in the TCGA cohort (n = 145).

Variable	EPV/n	Univariable Analysis				Multivariable Analysis ^#^			
		HR	95% CI		*p*	HR	95% CI		*p*
			Lower	Upper			Lower	Upper	
*SKA3* (low vs. high)	34/71_50/74	1.84	1.18	2.86	**0.007**	2.13	1.36	3.34	**<0.001**
Age (≤73 vs. >73)	59/107_25/38	1.63	1.01	2.63	**0.04**	1.73	1.07	2.79	**0.03**
Sex (female vs. grade)	43/68_41/77	0.82	0.53	1.26	0.36	-	-	-	-
Grade (G1–G2 vs. G3–G4)	56/103_28/42	1.38	0.87	2.17	0.17	-	-	-	-
pT (T1–T2 vs. T3–T4)	9/19_75/126	1.14	0.57	2.29	0.71	-	-	-	-
pN (absent vs. present)	17/37_67/108	1.51	0.88	2.57	0.13	-	-	-	-
TNM stage (I–II vs. III–IV)	81/138_3/7	0.57	0.18	1.80	0.34	-	-	-	-
Radiation Therapy (no vs. yes)	66/107_18/38								
Radiation Therapy		0.28 ^T^	0.10	0.81	**0.02**	0.28	0.10	0.79	**0.02**
Radiation Therapy*T_COV_		1.05 ^T^	0.99	1.11	0.10	1.04	0.98	1.10	0.17

Abbreviations: CI—confidence interval, EPV—events per variable, HR—hazard ratio, TCGA—The Cancer Genome Atlas. Significant *p*-values (p < 0.05) are indicated in bold. ^#^ Final result of a multivariable Cox regression model built of variables with *p*-value < 0.05 in univariable analysis. ^T^ HR for time-dependent variable.

## Data Availability

Publicly available datasets were analyzed in this study. These data can be found here: https://www.cbioportal.org/study/summary?id=paad_tcga_pan_can_atlas_2018 (accessed on 26 October 2023), https://xenabrowser.net (accessed on 26 October 2023), http://ualcan.path.uab.edu/ (accessed on 27 July 2022), https://reactome.org (accessed on 27 July 2022), https://www.genome.jp/kegg/brite.html (accessed on 27 July 2022), https://david.ncifcrf.gov (accessed on 27 July 2022), https://string-db.org (accessed on 27 July 2022). Our data presented in this study are available on request from the corresponding author. The data are not publicly available due to ethical restrictions.

## Supplementary Materials

The following supporting information can be downloaded at: https://www.mdpi.com/article/10.3390/ijms25105134/s1.
