# Supplementary material for: SKA3 Expression as a Prognostic Factor for Patients with Pancreatic Adenocarcinoma
Source: Int J Mol Sci. 2024 May 9;25(10):5134. doi: 10.3390/ijms25105134 (PMC11120893; doi:10.3390/ijms25105134)
Supplement: Supplementary file 1 [file ijms-25-05134-s001.zip › ijms-2973948-supplementary.pdf]

SUPPLEMENTARY MATERIAL

Table S1. Association of SKA3 protein expression in PDAC tumors with patients' characteristics (TMA cohort, n = 110).

**Clinicopathological feature**	n (%) n = 110	**SKA3 expression** low n = 71	high n = 39	***p***
Age (years)				
≤ 70	89 (80.9)	57 (64.0)	32 (36.0)	>0.99
> 70	21 (19.1)	14 (66.7)	7 (33.3)	
Sex				
Female	53 (48.2)	35 (66.0)	18 (34.0)	0.84
Male	57 (51.8)	36 (63.2)	21 (36.8)	
Grading				
G1	10 (9.1)	4 (40.0)	6 (60.0)	
G2	90 (81.8)	60 (66.7)	30 (33.3)	0.16
G3	10 (9.1)	7 (70.0)	3 (30.0)	
pT status				
T1-T2	78 (70.9)	52 (66.7)	26 (33.3)	0.51
T3-T4	32 (29.1)	19 (59.4)	13 (40.6)	
pN status				
N0	50 (45.5)	33 (66.0)	17 (34.0)	0.84
N1-N2	60 (54.5)	38 (63.3)	22 (36.7)	
pM status				
M0	100 (90.9)	63 (63.0)	37 (37.0)	0.49
M1	10 (9.1)	8 (80.0)	2 (20.0)	
TNM stage				
I-II	75 (68.2)	50 (66.7)	25 (33.3)	0.53
III-IV	35 (31.8)	21 (60.0)	14 (40.0)	
VI				
Absent	84 (76.4)	56 (66.7)	28 (33.3)	0.48
Present	26 (23.6)	15 (57.7)	11 (42.3)	
PNI				
Absent	21 (19.1)	14 (66.7)	7 (33.3)	>0.99
Present	89 (80.9)	57 (64.0)	32 (36.0)	
Resection margin				
R0	76 (69.1)	50 (65.8)	26 (34.2)	0.83
R1	34 (30.9)	21 (61.8)	13 (38.2)	
CTX				
No	27 (24.6)	17 (63.0)	10 (37.0)	>0.99
Yes	83 (75.4)	54 (65.1)	29 (34.9)	

Abbreviations: CTX – chemotherapy, VI – vascular invasion, PDAC – pancreatic ductal adenocarcinoma, PNI – perineural invasion, TMA – tissue macroarray. Chi-square or Fisher's exact test.

Table S2. Association of SKA3 mRNA expression in PDAC tumors with patients' characteristics (TCGA cohort, n = 145).

**Clinicopathological feature**	n (%) n = 145	***SKA3* expression**		***p***
		low n = 71	high n = 74	
Age (years)				
≤ 73	111 (76.55)	56 (50.45)	55 (49.55)	0.56
> 73	34 (23.45)	15 (44.12)	19 (55.88)	
Sex				
Female	68 (46.90)	34 (50.00)	34 (50.00)	0.87
Male	77 (53.10)	37 (48.05)	40 (51.95)	
Grading				
G1-G2	103 (71.03)	52 (50.49)	51 (49.51)	0.59
G3-G4	42 (28.97)	19 (45.24)	23 (54.76)	
pT status				
T1-T2	19 (13.10)	9 (47.37)	10 (52.63)	>0.99
T3-T4	125 (86.21)	62 (49.60)	63 (50.40)	
Missing	1 (0.69)	-	-	
pN status				
N0	37 (25.52)	18 (48.65)	19 (51.35)	>0.99
N1	107 (73.79)	52 (48.60)	55 (51.40)	
Missing	1 (0.69)	-	-	
pM status				
M0	69 (47.59)	37 (53.62)	32 (46.38)	>0.99
M1	3 (2.07)	2 (66.67)	1 (33.33)	
Missing	73 (50.34)	-	-	
TNM stage				
I	12 (8.27)	5 (41.67)	7 (58.33)	
II	126 (86.90)	62 (49.21)	64 (50.79)	0.35
III-IV	6 (4.14)	4 (66.67)	2 (33.33)	
Missing	1 (0.69)	-	-	
Radiation therapy				
No	95 (65.52)	48 (50.53)	47 (49.47)	0.44
Yes	36 (24.83)	15 (41.67)	21 (58.33)	
Missing	14 (9.65)			

Abbreviations: TCGA – The Cancer Genome Atlas.

Table S3. Reactome pathway enrichment analysis for the top 50 genes positively correlated with *SKA3* in pancreatic adenocarcinoma (PAAD).

Pathway	ID	Gene count	q-value	Genes
Cell Cycle	R-HSA-1640170	37	7,33E-15	ERCC6L;HJURP;NCAPG;BUB1B;CENPA;NCAPH;CCNB2;CCNB1;CDC45;SGOL1;EXO1;NUF2;RAD54L;BUB1;GTSE1;GINS1;UBE2C;PLK1;ORC6L;KIF23;ZWINT;TPX2;CENPF;RAD51;CDK1;BIRC5;CENPN;KIF2C;KIF20A
Cell Cycle, Mitotic	R-HSA-69278	32	7,33E-15	ERCC6L;NCAPG;BUB1B;CENPA;NCAPH;CCNB2;CCNB1;CDC45;SGOL1;NUF2;BUB1;GTSE1;GINS1;UBE2C;PLK1;ORC6L;KIF23;ZWINT;TPX2;CENPF;CDK1;BIRC5;CENPN;KIF2C;KIF20A
Cell Cycle Checkpoints	R-HSA-69620	21	7,33E-15	ERCC6L;UBE2C;PLK1;BUB1B;ORC6L;CENPA;ZWINT;CCNB2;CENPF;CCNB1;CDC45;SGOL1;EXO1;NUF2;CDK1;BIRC5;KIF2C;CENPN;GTSE1;BUB1
Mitotic Prometaphase	R-HSA-68877	17	7,33E-15	ERCC6L;PLK1;NCAPG;BUB1B;CENPA;NCAPH;ZWINT;CCNB2;CENPF;CCNB1;SGOL1;NUF2;CDK1;BIRC5;KIF2C;CENPN;BUB1
Resolution of Sister Chromatid Cohesion	R-HSA-2500257	15	7,33E-15	ERCC6L;PLK1;BUB1B;CENPA;ZWINT;CCNB2;CENPF;CCNB1;SGOL1;NUF2;CDK1;BIRC5;KIF2C;CENPN;BUB1
M Phase	R-HSA-68886	20	1,83E-14	ERCC6L;UBE2C;PLK1;NCAPG;BUB1B;KIF23;CENPA;NCAPH;ZWINT;CCNB2;CENPF;CCNB1;SGOL1;NUF2;CDK1;BIRC5;KIF2C;CENPN;KIF20A;BUB1
Mitotic Spindle Checkpoint	R-HSA-69618	13	7,83E-14	ERCC6L;UBE2C;PLK1;BUB1B;CENPA;ZWINT;CENPF;SGOL1;NUF2;BIRC5;KIF2C;CENPN;BUB1
Mitotic Anaphase	R-HSA-68882	16	2,40E-13	ERCC6L;UBE2C;PLK1;BUB1B;CENPA;ZWINT;CCNB2;CENPF;CCNB1;SGOL1;NUF2;CDK1;BIRC5;KIF2C;CENPN;BUB1
Mitotic Metaphase and Anaphase	R-HSA-2555396	16	2,40E-13	ERCC6L;UBE2C;PLK1;BUB1B;CENPA;ZWINT;CCNB2;CENPF;CCNB1;SGOL1;NUF2;CDK1;BIRC5;KIF2C;CENPN;BUB1
Amplification of signal from unattached kinetochores via a MAD2 inhibitory signal	R-HSA-141444	12	2,40E-13	ERCC6L;CENPF;SGOL1;PLK1;NUF2;BIRC5;BUB1B;KIF2C;CENPN;BUB1;CENPA;ZWINT
Amplification of signal from the kinetochores	R-HSA-141424	12	2,40E-13	ERCC6L;CENPF;SGOL1;PLK1;NUF2;BIRC5;BUB1B;KIF2C;CENPN;BUB1;CENPA;ZWINT
RHO GTPases Activate Formins	R-HSA-5663220	13	1,80E-12	ERCC6L;PLK1;BUB1B;CENPA;ZWINT;CENPF;DIAPH3;SGOL1;NUF2;BIRC5;KIF2C;CENPN;BUB1
Polo-like kinase mediated events	R-HSA-156711	8	2,91E-12	CCNB2;CCNB1;CENPF;PLK1
EML4 and NUDC in mitotic spindle formation	R-HSA-9648025	12	3,49E-12	ERCC6L;CENPF;SGOL1;PLK1;NUF2;BIRC5;BUB1B;KIF2C;CENPN;BUB1;CENPA;ZWINT
Separation of Sister Chromatids	R-HSA-2467813	13	4,20E-11	ERCC6L;UBE2C;PLK1;BUB1B;CENPA;ZWINT;CENPF;SGOL1;NUF2;BIRC5;KIF2C;CENPN;BUB1
G2/M Transition	R-HSA-69275	12	1,90E-09	CCNB2;TPX2;CCNB1;CENPF;PLK1;CDK1;GTSE1
Mitotic G2-G2/M phases	R-HSA-453274	12	2,00E-09	CCNB2;TPX2;CCNB1;CENPF;PLK1;CDK1;GTSE1
Kinesins	R-HSA-983189	8	1,05E-08	KIF18B;KIFC1;KIF4A;KIF2C;KIF23;KIF20A;KIF11;KIF15
RHO GTPase Effectors	R-HSA-195258	13	1,71E-08	ERCC6L;PLK1;BUB1B;CENPA;ZWINT;CENPF;DIAPH3;SGOL1;NUF2;BIRC5;KIF2C;CENPN;BUB1
Condensation of Prometaphase Chromosomes	R-HSA-2514853	5	1,11E-07	CCNB2;CCNB1;CDK1;NCAPG;NCAPH
COPI-dependent Golgi-to-ER retrograde traffic	R-HSA-6811434	8	2,90E-07	KIF18B;KIFC1;KIF4A;KIF2C;KIF23;KIF20A;KIF11;KIF15
Activation of NIMA Kinases NEK9, NEK6, NEK7	R-HSA-2980767	4	1,16E-06	CCNB2;CCNB1;PLK1;CDK1
Golgi-to-ER retrograde transport	R-HSA-8856688	8	3,17E-06	KIF18B;KIFC1;KIF4A;KIF2C;KIF23;KIF20A;KIF11;KIF15
Cyclin A/B1/B2 associated events during G2/M transition	R-HSA-69273	5	3,77E-06	CCNB2;CCNB1;PLK1;CDK1
G2/M Checkpoints	R-HSA-69481	8	3,78E-06	CCNB2;CCNB1;CDC45;UBE2C;EXO1;CDK1;ORC6L;GTSE1
Golgi Cisternae Pericentriolar Stack Reorganization	R-HSA-162658	4	1,15E-05	CCNB2;CCNB1;PLK1;CDK1
Regulation of APC/C activators between G1/S and early anaphase	R-HSA-176408	6	1,86E-05	CCNB1;UBE2C;PLK1;CDK1;BUB1B;BUB1
Factors involved in megakaryocyte development and platelet production	R-HSA-983231	8	1,87E-05	KIF18B;KIFC1;KIF4A;KIF2C;KIF23;KIF20A;KIF11;KIF15
Phosphorylation of the APC/C	R-HSA-176412	4	2,00E-05	CCNB1;UBE2C;PLK1;CDK1
APC/C-mediated degradation of cell cycle proteins	R-HSA-174143	6	2,79E-05	CCNB1;UBE2C;PLK1;CDK1;BUB1B;BUB1
Regulation of mitotic cell cycle	R-HSA-453276	6	2,79E-05	CCNB1;UBE2C;PLK1;CDK1;BUB1B;BUB1
G1/S Transition	R-HSA-69206	7	3,55E-05	CCNB1;CDC45;CDK1;ORC6L
G2/M DNA replication checkpoint	R-HSA-69478	3	3,71E-05	CCNB2;CCNB1;CDK1
Intra-Golgi and retrograde Golgi-to-ER traffic	R-HSA-6811442	8	3,71E-05	KIF18B;KIFC1;KIF4A;KIF2C;KIF23;KIF20A;KIF11;KIF15
Phosphorylation of Emi1	R-HSA-176417	3	5,40E-05	CCNB1;PLK1;CDK1
Signaling by Rho GTPases	R-HSA-194315	13	6,47E-05	ERCC6L;PLK1;BUB1B;CENPA;ZWINT;CENPF;DIAPH3;SGOL1;NUF2;BIRC5;KIF2C;CENPN;BUB1
E2F-enabled inhibition of pre-replication complex formation	R-HSA-113507	3	7,29E-05	CCNB1;CDK1;ORC6L
Signaling by Rho GTPases, Miro GTPases and RHOBTB3	R-HSA-9716542	13	7,29E-05	ERCC6L;PLK1;BUB1B;CENPA;ZWINT;CENPF;DIAPH3;SGOL1;NUF2;BIRC5;KIF2C;CENPN;BUB1
Mitotic G1 phase and G1/S transition	R-HSA-453279	7	7,41E-05	CCNB1;CDC45;CDK1;ORC6L
Activation of APC/C and APC/C:Cdc20 mediated degradation of mitotic proteins	R-HSA-176814	5	1,62E-04	CCNB1;UBE2C;PLK1;CDK1;BUB1B;BUB1
The role of GTSE1 in G2/M progression after G2 checkpoint	R-HSA-8852276	5	2,31E-04	CCNB2;CCNB1;PLK1;CDK1;GTSE1
Transcriptional Regulation by TP53	R-HSA-3700989	10	2,43E-04	FANCI;TPX2;CCNB1;EXO1;CDK1;BIRC5

G1/S-Specific Transcription	R-HSA-69205	4	2,52E-04	CDC45;CDK1
Mitotic Telophase/Cytokinesis	R-HSA-68884	3	2,71E-04	PLK1;KIF23;KIF20A
MHC class II antigen presentation	R-HSA-2132295	6	2,92E-04	KIF4A;KIF2C;KIF23;KIF20A;KIF11;KIF15
APC/C:Cdc20 mediated degradation of Cyclin B	R-HSA-174048	3	1,05E-03	CCNB1;UBE2C;CDK1
E2F mediated regulation of DNA replication	R-HSA-113510	3	1,05E-03	CCNB1;CDK1;ORC6L
Nuclear Envelope Breakdown	R-HSA-2980766	4	1,05E-03	CCNB2;CCNB1;PLK1;CDK1
Initiation of Nuclear Envelope (NE) Reformation	R-HSA-2995383	3	1,31E-03	CCNB2;CCNB1;CDK1
Synthesis of DNA	R-HSA-69239	5	1,50E-03	GINS1;CDC45;UBE2C;CDK1;ORC6L
Chromosome Maintenance	R-HSA-73886	5	1,72E-03	HJURP;CDK1;RAD54L;CENPN;CENPA
APC/C:Cdc20 mediated degradation of mitotic proteins	R-HSA-176409	4	1,90E-03	CCNB1;UBE2C;CDK1;BUB1B;BUB1
G2/M DNA damage checkpoint	R-HSA-69473	4	2,31E-03	CCNB1;UBE2C;EXO1;CDK1
Diseases of mitotic cell cycle	R-HSA-9675126	3	3,24E-03	UBE2C;CDK1;RAD54L
Regulation of PLK1 Activity at G2/M Transition	R-HSA-2565942	4	3,24E-03	CCNB2;CCNB1;PLK1;CDK1
DNA Replication	R-HSA-69306	5	3,82E-03	GINS1;CDC45;UBE2C;CDK1;ORC6L
Nuclear Pore Complex (NPC) Disassembly	R-HSA-3301854	3	3,92E-03	CCNB2;CCNB1;CDK1
S Phase	R-HSA-69242	5	4,93E-03	GINS1;CDC45;UBE2C;CDK1;ORC6L
DNA Repair	R-HSA-73894	7	5,65E-03	FANCI;RAD51;UBE2C;EXO1;UBE2T;CDK1;DTL
Unwinding of DNA	R-HSA-176974	2	6,13E-03	GINS1;CDC45
MASTL Facilitates Mitotic Progression	R-HSA-2465910	2	6,13E-03	CCNB1;CDK1
Deposition of new CENPA-containing nucleosomes at the centromere	R-HSA-606279	3	7,60E-03	HJURP;CENPN;CENPA
Nucleosome assembly	R-HSA-774815	3	7,60E-03	HJURP;CENPN;CENPA
Condensation of Prophase Chromosomes	R-HSA-2299718	3	7,60E-03	CCNB1;PLK1;CDK1
Chk1/Chk2(Cds1) mediated inactivation of Cyclin B:Cdk1 complex	R-HSA-75035	2	7,60E-03	CCNB1;CDK1
HDR through Homologous Recombination (HRR) or Single Strand Annealing (SSA)	R-HSA-5693567	4	7,73E-03	RAD51;UBE2C;EXO1;CDK1
Homology Directed Repair	R-HSA-5693538	4	9,16E-03	RAD51;UBE2C;EXO1;CDK1
Mitotic Prophase	R-HSA-68875	4	1,05E-02	CCNB2;CCNB1;PLK1;CDK1
Transcription of E2F targets under negative control by p107 (RBL1) and p130 (RBL2) in complex with HDAC1	R-HSA-1362300	2	1,33E-02	CDK1
Inactivation of APC/C via direct inhibition of the APC/C complex	R-HSA-141430	2	1,46E-02	UBE2C;BUB1B;BUB1
Inhibition of the proteolytic activity of APC/C required for the onset of anaphase by mitotic spindle checkpoint components	R-HSA-141405	2	1,46E-02	UBE2C;BUB1B;BUB1
TP53 Regulates Transcription of Genes Involved in G2 Cell Cycle Arrest	R-HSA-6804114	2	1,46E-02	CCNB1;CDK1
Cdc20:Phospho-APC/C mediated degradation of Cyclin A	R-HSA-174184	3	1,50E-02	UBE2C;CDK1;BUB1B;BUB1
APC:Cdc20 mediated degradation of cell cycle proteins prior to satisfation of the cell cycle checkpoint	R-HSA-179419	3	1,54E-02	UBE2C;CDK1;BUB1B;BUB1
AURKA Activation by TPX2	R-HSA-8854518	3	1,54E-02	TPX2;PLK1;CDK1
Depolymerisation of the Nuclear Lamina	R-HSA-4419969	2	1,54E-02	CCNB1;CDK1
Impaired BRCA2 binding to PALB2	R-HSA-9709603	2	1,54E-02	RAD51;EXO1
Generic Transcription Pathway	R-HSA-212436	14	1,54E-02	FANCI;TPX2;CCNB1;RAD51;UBE2C;EXO1;CDK1;BIRC5;CEP55
Defective HDR through Homologous Recombination (HRR) due to PALB2 loss of function	R-HSA-9701193	2	1,54E-02	RAD51;EXO1
Defective HDR through Homologous Recombination Repair (HRR) due to PALB2 loss of BRCA2/RAD51/RAD51C binding function	R-HSA-9704646	2	1,54E-02	RAD51;EXO1
Defective HDR through Homologous Recombination Repair (HRR) due to PALB2 loss of BRCA1 binding function	R-HSA-9704331	2	1,54E-02	RAD51;EXO1
Defective HDR through Homologous Recombination (HRR) due to BRCA1 loss-of-function	R-HSA-9701192	2	1,54E-02	RAD51;EXO1
Protein ubiquitination	R-HSA-8852135	3	1,66E-02	UBE2C;UBE2T
APC-Cdc20 mediated degradation of Nek2A	R-HSA-179409	2	1,66E-02	UBE2C;BUB1B;BUB1
DNA Double-Strand Break Repair	R-HSA-5693532	4	1,74E-02	RAD51;UBE2C;EXO1;CDK1
TP53 Regulates Transcription of DNA Repair Genes	R-HSA-6796648	3	1,77E-02	FANCI
Nuclear Envelope (NE) Reassembly	R-HSA-2995410	3	1,88E-02	CCNB2;CCNB1;CDK1
TP53 regulates transcription of several additional cell death genes whose specific roles in p53-dependent apoptosis remain uncertain	R-HSA-6803205	2	1,92E-02	BIRC5

Processing of DNA double-strand break ends	R-HSA-5693607	3	1,94E-02	UBE2C;EXO1;CDK1
Regulation of TP53 Activity	R-HSA-5633007	4	2,08E-02	TPX2;EXO1;CDK1
Membrane Trafficking	R-HSA-199991	8	2,09E-02	KIF18B;KIFC1;KIF4A;KIF2C;KIF23;KIF20A;KIF11;KIF15
Switching of origins to a post-replicative state	R-HSA-69052	3	2,09E-02	UBE2C;CDK1;ORC6L
Regulation of TP53 Activity through Phosphorylation	R-HSA-6804756	3	2,09E-02	TPX2;EXO1;CDK1
Resolution of D-loop Structures through Synthesis-Dependent Strand Annealing (SDSA)	R-HSA-5693554	2	2,09E-02	RAD51;EXO1
Defective Inhibition of DNA Recombination at Telomere	R-HSA-9670621	1	2,09E-02	RAD54L
Alternative Lengthening of Telomeres (ALT)	R-HSA-9006821	1	2,09E-02	RAD54L
Diseases of Telomere Maintenance	R-HSA-9673013	1	2,09E-02	RAD54L
Defective Inhibition of DNA Recombination at Telomere Due to ATRX Mutations	R-HSA-9670615	1	2,09E-02	RAD54L
Defective Inhibition of DNA Recombination at Telomere Due to DAXX Mutations	R-HSA-9670613	1	2,09E-02	RAD54L
Synthesis of active ubiquitin: roles of E1 and E2 enzymes	R-HSA-8866652	2	2,09E-02	UBE2C;UBE2T
Gene expression (Transcription)	R-HSA-74160	15	2,09E-02	FANCI;TPX2;CCNB1;RAD51;UHRF1;UBE2C;EXO1;CDK1;BIRC5;CEP55
Impaired BRCA2 binding to RAD51	R-HSA-9709570	2	2,09E-02	RAD51;EXO1
Activation of the pre-replicative complex	R-HSA-68962	2	2,09E-02	CDC45;ORC6L
Aberrant regulation of mitotic cell cycle due to RB1 defects	R-HSA-9687139	2	2,09E-02	UBE2C;CDK1
NOTCH3 Intracellular Domain Regulates Transcription	R-HSA-9013508	2	2,09E-02	DLGAP5
RNA Polymerase II Transcription	R-HSA-73857	14	2,09E-02	FANCI;TPX2;CCNB1;RAD51;UBE2C;EXO1;CDK1;BIRC5;CEP55
Resolution of D-loop Structures through Holliday Junction Intermediates	R-HSA-5693568	2	2,18E-02	RAD51;EXO1
Resolution of D-Loop Structures	R-HSA-5693537	2	2,29E-02	RAD51;EXO1
DNA strand elongation	R-HSA-69190	2	2,29E-02	GINS1;CDC45
G0 and Early G1	R-HSA-1538133	2	2,29E-02	CDK1
HDR through Single Strand Annealing (SSA)	R-HSA-5685938	2	2,41E-02	RAD51;EXO1
Activation of ATR in response to replication stress	R-HSA-176187	2	2,41E-02	CDC45;ORC6L
Diseases of DNA Double-Strand Break Repair	R-HSA-9675136	2	2,64E-02	RAD51;EXO1
Defective homologous recombination repair (HRR) due to BRCA2 loss of function	R-HSA-9701190	2	2,64E-02	RAD51;EXO1
Presynaptic phase of homologous DNA pairing and strand exchange	R-HSA-5693616	2	2,77E-02	RAD51;EXO1
Regulation of TP53 Degradation	R-HSA-6804757	2	2,89E-02	CDK1
Homologous DNA Pairing and Strand Exchange	R-HSA-5693579	2	3,15E-02	RAD51;EXO1
Transcriptional Regulation by E2F6	R-HSA-8953750	2	3,28E-02	RAD51
Regulation of TP53 Expression and Degradation	R-HSA-6806003	2	3,28E-02	CDK1
Mitotic Metaphase/Anaphase Transition	R-HSA-68881	1	3,35E-02	PLK1
DNA Replication Pre-Initiation	R-HSA-69002	3	3,72E-02	CDC45;UBE2C;ORC6L
Hemostasis	R-HSA-109582	8	3,91E-02	KIF18B;KIFC1;KIF4A;KIF2C;KIF23;KIF20A;KIF11;KIF15
Phosphorylation of proteins involved in the G2/M transition by Cyclin A:Cdc2 complexes	R-HSA-170145	1	4,18E-02	CDK1
Fanconi Anemia Pathway	R-HSA-6783310	2	4,28E-02	FANCI;UBE2T
Diseases of DNA repair	R-HSA-9675135	2	4,43E-02	RAD51;EXO1
Vesicle-mediated transport	R-HSA-5653656	8	4,61E-02	KIF18B;KIFC1;KIF4A;KIF2C;KIF23;KIF20A;KIF11;KIF15
Phosphorylation of proteins involved in G1/S transition by active Cyclin E:Cdk2 complexes	R-HSA-69200	1	5,01E-02	CDK1

Table S4. Kyoto Encyclopedia of Genes and Genomes (KEGG) pathway analysis for the top 50 genes positively correlated with *SKA3* in PAAD.

Pathway	ID	Gene count	q-value	Genes
Cell cycle	hsa04110	9	4,25E-09	CCNB2, CCNB1, ORC6, CDC45, PLK1, CDK1, BUB1B, TTK, BUB1
Oocyte meiosis	hsa04114	6	1,20E-04	SGO1, CCNB2, CCNB1, PLK1, CDK1, BUB1
Progesterone-mediated oocyte maturation	hsa04914	5	6,45E-04	CCNB2, CCNB1, PLK1, CDK1, BUB1
p53 signaling pathway	hsa04115	4	3,76E-03	CCNB2, CCNB1, CDK1, GTSE1
Fanconi anemia pathway	hsa03460	3	3,42E-02	FANCI, RAD51, UBE2T
FoxO signaling pathway	hsa04068	3	1,53E-01	CCNB2, CCNB1, PLK1
Cellular senescence	hsa04218	3	1,79E-01	CCNB2, CCNB1, CDK1
Human immunodeficiency virus 1 infection	hsa05170	3	2,54E-01	CCNB2, CCNB1, CDK1
Homologous recombination	hsa03440	2	2,54E-01	RAD51, RAD54L

Table S5. Gene ontology (GO) enrichment analysis for the *SKA3* and the top 50 co-upregulated genes in PAAD.

GO Terms	ID	Gene count	q-value	Genes
**Biological process**				
Cell division	GO:0051301	23	1,62E-22	ERCC6L, CDCA2, SPAG5, UBE2C, NCAPG, BUB1B, KIF11, PIMREG, SKA3, NCAPH, ZWINT, SGO1, CCNB2, TPX2, CENPF, CCNB1, KIF18B, KIFC1, NUF2, CDK1, BIRC5, KIF2C, BUB1
Chromosome segregation	GO:0007059	11	5,04E-13	SGO1, CENPF, CDCA2, SPAG5, NUF2, HJURP, BIRC5, TTK, CENPN, SKA3, DLGAP5
Mitotic spindle assembly checkpoint	GO:0007094	8	1,34E-10	CENPF, PLK1, NUF2, BIRC5, BUB1B, TTK, BUB1, ZWINT
Mitotic sister chromatid segregation	GO:0000070	8	1,99E-10	SGO1, KIF18B, SPAG5, KIFC1, PLK1, NUSAP1, KIF11, ZWINT
Mitotic spindle organization	GO:0007052	8	5,28E-09	CCNB1, KIF4A, PLK1, NUF2, BIRC5, TTK, KIF11, DLGAP5
Mitotic cytokinesis	GO:0000281	8	1,61E-08	KIF4A, PLK1, NUSAP1, BIRC5, KIF23, KIF20A, CENPA, CEP55
Microtubule-based movement	GO:0007018	8	1,05E-07	KIF18B, KIFC1, KIF4A, KIF23, KIF2C, KIF20A, KIF11, KIF15
Mitotic cell cycle	GO:0000278	9	1,68E-07	TPX2, CENPF, KIF18B, PLK1, BIRC5, BUB1B, KIF11, SKA3, KIF15
Cell cycle	GO:0007049	10	8,24E-06	FANCI, ERCC6L, CDCA2, CDC45, UHRF1, NUF2, HJURP, BIRC5, KIF11, PIMREG
Kinetochore assembly	GO:0051382	4	1,95E-04	CENPF, CENPN, CENPA, DLGAP5
Spindle organization	GO:0007051	4	2,64E-04	ASPM, SPAG5, TTK, KIF11
Mitotic chromosome condensation	GO:0007076	4	2,90E-04	PLK1, NUSAP1, NCAPG, NCAPH
DNA repair	GO:0006281	7	2,11E-03	FANCI, RAD51, EXO1, UHRF1, UBE2T, CDK1, RAD54L
Mitotic cell cycle checkpoint	GO:0007093	3	3,56E-03	BUB1B, BUB1, ZWINT
Mitotic spindle midzone assembly	GO:0051256	3	6,49E-03	KIF4A, BIRC5, KIF23
Regulation of chromosome segregation	GO:0051983	3	7,29E-03	CDCA2, KIF2C, BUB1
G2/M transition of mitotic cell cycle	GO:0000086	4	7,97E-03	CCNB1, PLK1, CDK1, BIRC5
DNA unwinding involved in DNA replication	GO:0006268	3	2,44E-02	GINS1, RAD51, CDC45
Positive regulation of G2/M transition of mitotic cell cycle	GO:0010971	3	3,67E-02	CCNB1, CDK1, DTL
**Cellular components**				
Kinetochore	GO:0000776	16	1,34E-18	ERCC6L, SPAG5, PLK1, HJURP, BUB1B, TTK, SKA3, CENPA, ZWINT, SGO1, CENPF, NUF2, BIRC5, KIF2C, CENPN, BUB1
Spindle	GO:0005819	12	2,30E-12	ASPM, TPX2, CENPF, PLK1, NUSAP1, BUB1B, KIF23, TTK, KIF2C, KIF20A, KIF11, KIF15
Nucleoplasm	GO:0005654	33	1,38E-10	CDCA2, UHRF1, HJURP, CENPA, NCAPH, CCNB1, ORC6, CDC45, EXO1, NUF2, RAD54L, GTSE1, BUB1, GINS1, FANCI, UBE2C, PLK1, KIF23, PIMREG, ZWINT, SGO1, TPX2, CENPF, RAD51, KIF18B, KIF4A, UBE2T, CDK1, BIRC5, CENPN, KIF20A, DTL, ASF1B
Centrosome	GO:0005813	14	8,93E-09	PLK1, KIF23, SKA3, KIF15, SGO1, ASPM, CCNB2, CENPF, CCNB1, CDC45, CDK1, KIF2C, DTL, CEP55
Midbody	GO:0030496	10	9,75E-09	ASPM, CENPF, SPAG5, KIF4A, PLK1, CDK1, BIRC5, KIF23, KIF20A, CEP55
Kinesin complex	GO:0005871	7	3,93E-08	KIF18B, KIFC1, KIF23, KIF2C, KIF20A, KIF11, KIF15
Cytosol	GO:0005829	34	8,46E-08	ERCC6L, CDCA2, NCAPG, BUB1B, KIF11, SKA3, CENPA, NCAPH, KIF15, CCNB2, CCNB1, ORC6, NUF2, GTSE1, BUB1, DLGAP5, FANCI, SPAG5, UBE2C, PLK1, KIF23, ZWINT, SGO1, TPX2, CENPF, DIAPH3, KIF18B, KIF4A, CDK1, BIRC5, CENPN, KIF2C, DTL, FAM72B
Chromosome, centromeric region	GO:0000775	7	9,63E-08	SGO1, CENPF, NUF2, HJURP, BIRC5, KIF2C, CENPA
Condensed chromosome outer kinetochore	GO:0000940	5	1,38E-07	CENPF, CCNB1, PLK1, BUB1B, SKA3
Mitotic spindle	GO:0072686	8	3,81E-07	TPX2, SPAG5, KIFC1, NUSAP1, CDK1, KIF23, KIF11, SKA3
Spindle microtubule	GO:0005876	6	5,27E-07	KIF4A, PLK1, CDK1, BIRC5, KIF11, SKA3
Microtubule	GO:0005874	10	5,62E-07	TPX2, KIF18B, KIFC1, KIF4A, NUSAP1, KIF23, KIF2C, KIF20A, KIF11, KIF15
Nucleus	GO:0005634	33	1,77E-06	CDCA2, UHRF1, HJURP, NCAPG, TTK, KIF11, CENPA, CCNB2, CCNB1, CDC45, EXO1, NUSAP1, RAD54L, DLGAP5, GINS1, UBE2C, PLK1, KIF23, ASPM, TPX2, CENPF, RAD51, DIAPH3, KIF18B, KIFC1, UBE2T, CDK1, BIRC5, KIF2C, KIF20A, HROB, DTL, ASF1B
Spindle pole	GO:0000922	6	8,41E-05	SGO1, TPX2, CENPF, CCNB1, PLK1, KIF11
Cytoplasm	GO:0005737	29	8,41E-05	NCAPG, BUB1B, TTK, KIF11, NCAPH, KIF15, CCNB2, CCNB1, CDC45, NUSAP1, DLGAP5, CEP55, GINS1, FANCI, SPAG5, PLK1, KIF23, ZWINT, ASPM, TPX2, CENPF, RAD51, DIAPH3, KIF18B, KIFC1, KIF4A, CDK1, BIRC5, KIF2C
Membrane	GO:0016020	19	8,41E-05	FANCI, ERCC6L, NCAPG, TTK, KIF11, NCAPH, KIF15, CCNB2, CCNB1, ORC6, KIFC1, KIF4A, NUF2, CDK1, KIF2C, GTSE1, BUB1, FAM72B, CEP55
Microtubule cytoskeleton	GO:0015630	6	5,57E-04	CCNB2, TPX2, PLK1, BIRC5, KIF2C, GTSE1
Condensed chromosome, centromeric region	GO:0000779	3	3,09E-03	SGO1, NCAPG, CENPA
Microtubule plus-end	GO:0035371	3	6,10E-03	KIF18B, SPAG5, KIF2C
Centriolar satellite	GO:0034451	4	1,19E-02	SPAG5, PLK1, DLGAP5, CEP55

Condensed nuclear chromosome	GO:0000794	3	1,42E-02	RAD51, NCAPG, NCAPH
Mitotic spindle pole	GO:0097431	3	1,43E-02	ASPM, SPAG5, PLK1
Cyclin-dependent protein kinase holoenzyme complex	GO:0000307	3	1,51E-02	CCNB2, CCNB1, CDK1
Cyclin B1-CDK1 complex	GO:0097125	2	1,65E-02	CCNB1, CDK1
Cytoplasmic microtubule	GO:0005881	3	2,87E-02	BIRC5, KIF2C, GTSE1
**Molecular function**				
Microtubule binding	GO:0008017	16	3,20E-15	SPAG5, PLK1, KIF23, KIF11, KIF15, TPX2, CENPF, KIF18B, KIFC1, KIF4A, NUSAP1, BIRC5, KIF2C, KIF20A, GTSE1, DLGAP5
ATP-dependent microtubule motor activity	GO:1990939	8	3,20E-10	KIF18B, KIFC1, KIF4A, KIF23, KIF2C, KIF20A, KIF11, KIF15
Microtubule motor activity	GO:0003777	8	2,95E-09	KIF18B, KIFC1, KIF4A, KIF23, KIF2C, KIF20A, KIF11, KIF15
ATP binding	GO:0005524	18	1,09E-06	ERCC6L, UBE2C, PLK1, BUB1B, TTK, KIF23, KIF11, KIF15, RAD51, KIF18B, KIFC1, UBE2T, KIF4A, CDK1, RAD54L, KIF2C, KIF20A, BUB1
Protein binding	GO:0005515	44	2,64E-04	ERCC6L, UHRF1, HJURP, NCAPG, BUB1B, TTK, KIF11, SKA3, CENPA, NCAPH, KIF15, CCNB2, CCNB1, ORC6, CDC45, EXO1, NUF2, NUSAP1, RAD54L, GTSE1, BUB1, DLGAP5, CEP55, FANCI, SPAG5, UBE2C, PLK1, KIF23, PIMREG, ZWINT, SGO1, TPX2, CENPF, RAD51, KIF18B, KIF4A, UBE2T, CDK1, BIRC5, KIF2C, KIF20A, HROB, DTL, ASF1B
ATPase activity	GO:0016887	8	2,76E-04	ERCC6L, KIF18B, KIFC1, RAD54L, KIF23, KIF2C, KIF20A, KIF15
Chromatin binding	GO:0003682	8	1,43E-03	CENPF, RAD51, CDC45, EXO1, UBE2T, CDK1, CENPA, NCAPH
DNA binding	GO:0003677	11	8,87E-03	FANCI, ERCC6L, RAD51, ORC6, EXO1, UHRF1, KIF4A, NUSAP1, HJURP, RAD54L, CENPA

Table S6. PPI network parameters computed with NetworkAnalyzer Cytoscape plugin.

Gene	BetweennessCentrality	ClosenessCentrality	ClusteringCoefficient	Degree
ASPM	1.40E-02	0.980	0.902	49
BUB1	1.40E-02	0.980	0.902	49
DLGAP5	1.94E-03	0.943	0.936	48
NCAPG	1.94E-03	0.943	0.936	48
CCNB1	1.94E-03	0.943	0.936	48
KIF23	1.94E-03	0.943	0.936	48
KIF11	1.94E-03	0.943	0.936	48
NUF2	1.94E-03	0.943	0.936	48
TTK	1.27E-02	0.962	0.918	48
CENPF	1.94E-03	0.943	0.936	48
CEP55	1.94E-03	0.943	0.936	48
BIRC5	1.94E-03	0.943	0.936	48
CDK1	1.94E-03	0.943	0.936	48
NUSAP1	1.94E-03	0.943	0.936	48
UBE2C	1.27E-02	0.962	0.918	48
KIF2C	1.27E-02	0.962	0.918	48
BUB1B	1.94E-03	0.943	0.936	48
KIF20A	1.94E-03	0.943	0.936	48
CDC45	1.94E-03	0.943	0.936	48
CCNB2	1.94E-03	0.943	0.936	48
ASF1B	1.43E-03	0.926	0.950	47
KIF15	1.21E-03	0.926	0.954	47
PLK1	1.43E-03	0.926	0.950	47
CENPA	1.30E-02	0.943	0.909	47
KIF4A	1.21E-03	0.926	0.954	47
TPX2	1.21E-03	0.926	0.954	47
SPAG5	1.64E-03	0.926	0.944	47
NCAPH	6.99E-04	0.909	0.969	46
CDCA2	9.17E-04	0.909	0.963	46
SKA3	9.17E-04	0.909	0.963	46
EXO1	6.99E-04	0.909	0.969	46
DTL	1.62E-03	0.909	0.943	46
ZWINT	5.91E-04	0.893	0.973	45
RAD54L	1.04E-03	0.893	0.962	45
HJURP	7.29E-04	0.893	0.970	45
ERCC6L	4.51E-04	0.877	0.978	44
FANCI	5.32E-04	0.877	0.975	44
FAM64A	3.17E-04	0.862	0.983	43
GTSE1	2.21E-04	0.862	0.988	43
UHRF1	9.70E-04	0.862	0.961	43
RAD51	4.68E-04	0.862	0.977	43
CENPN	3.12E-04	0.862	0.983	43
KIFC1	3.50E-04	0.847	0.981	42
KIF18B	3.74E-04	0.847	0.980	42
SGOL1	2.00E-04	0.833	0.988	41
UBE2T	3.98E-05	0.794	0.997	38
GINS1	5.52E-04	0.769	0.970	36
DIAPH3	2.86E-04	0.704	0.979	30
ORC6	2.29E-04	0.667	0.972	26
FAM72B	4.00E-02	0.538	0.714	7
C17orf53	0.00E+00	0.352	0.000	1

Table S7. Missing data on testing variables (TCGA cohort, n = 145).

Variable	Missing data n (%)
pT	1 (0.7)
pN	1 (0.7)
pM	73 (50.3)
TNM stage	1 (0.7)
Radiation therapy	14 (9.7)

Abbreviations: TCGA - The Cancer Genome Atlas

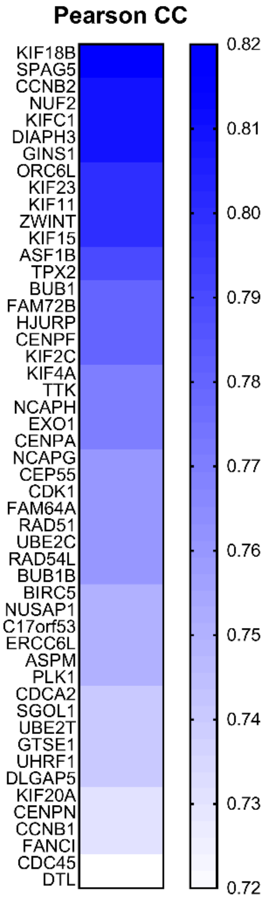

Figure S1. Top 50 genes positively correlated with *SKA3* in PDAC.
